# Mesenchymal stem cell-based therapy for osteoarthritis: a systematic review and meta-analysis of clinical outcomes and functional recovery

**DOI:** 10.3389/fcell.2025.1746471

**Published:** 2026-01-06

**Authors:** Jincheng Wang, Hao Xue, Mingfei Shi, Renshou Chen

**Affiliations:** 1 Institute of Literature in Chinese Medicine, Nanjing University of Chinese Medicine, Nanjing, China; 2 Jiangsu Provincial Research Institute of Chinese Medicine Schools, Nanjing, China; 3 School of Integrated Chinese and Western Medicine, Nanjing University of Chinese Medicine, Nanjing, China

**Keywords:** cartilage repair, cell therapy, functional recovery, joint preservation, mesenchymal stem cells, meta-analysis, osteoarthritis, pain management

## Abstract

**Backgrounds:**

This study aimed to evaluate the efficacy and safety of mesenchymal stem cell (MSC) therapy for osteoarthritis (OA) through a systematic review and meta-analysis of randomized controlled trials (RCTs), focusing on patient-reported pain and functional outcomes.

**Methods:**

A comprehensive literature search was conducted across multiple databases including PubMed, Embase, Web of Science, and Cochrane Library from inception to 1st October 2025. RCTs comparing intra-articular MSC injections with control interventions (placebo, hyaluronic acid, or other active treatments) in adult OA patients were included. Primary outcomes were changes in pain intensity measured by Visual Analog Scale (VAS) and functional improvement assessed by International Knee Documentation Committee (IKDC) score. Secondary outcomes included Western Ontario and McMaster Universities Osteoarthritis Index (WOMAC), Lequesne index, Lysholm score, and Tegner activity scale. Data were pooled using random-effects models and expressed as mean differences (MD) with 95% confidence intervals (CI).

**Results:**

Eleven RCTs involving 811 patients were included. MSC therapy demonstrated significant reduction in VAS pain scores compared to controls (MD -4.08, 95% CI -5.56 to −2.61, p < 0.00001), with the most pronounced effects at 24-month follow-up (MD -3.31, 95% CI -5.18 to −1.44, p = 0.0005). Significant improvements were observed in IKDC scores (MD 2.88, 95% CI 0.28 to 5.47, p = 0.03), WOMAC index (MD -11.05, 95% CI -15.97 to −6.14, p < 0.0001), Lequesne index (MD -5.32, 95% CI -5.91 to −4.74, p < 0.00001), Lysholm score (MD 5.07, 95% CI 1.86 to 8.29, p = 0.002), and Tegner activity scale (MD 0.44, 95% CI 0.25 to 0.62, p < 0.00001). The therapeutic effects showed a time-dependent pattern, with maximal benefits observed at 24-month follow-up across all outcome measures.

**Conclusion:**

Intra-articular MSC injection is an effective treatment for osteoarthritis, providing significant and durable improvements in pain relief, functional recovery, and activity levels up to 24 months post-treatment. The time-dependent nature of clinical benefits suggests a potential disease-modifying mechanism of action. MSC therapy represents a promising regenerative approach for OA management that warrants further investigation in large-scale trials.

## 1 Introduction

Osteoarthritis (OA) stands as the most prevalent degenerative joint disease and a leading cause of chronic pain and disability worldwide, imposing a staggering burden on healthcare systems and society (Trounson and McDonald, 2015). Traditionally viewed merely as a consequence of “wear and tear,” our understanding of OA pathogenesis has evolved to recognize it as a complex, multifactorial process involving not only the progressive degradation of articular cartilage but also synovial inflammation, subchondral bone remodeling, meniscal damage, and ligamentous changes (Yang et al., 2024a). This whole-joint pathology is driven by a interplay of mechanical, inflammatory, and metabolic factors, leading to an imbalance between catabolic and anabolic activities within the joint microenvironment (Zhu et al., 2022). The current therapeutic arsenal for OA remains predominantly palliative, structured in a stepwise manner. Initial management relies on non-pharmacological interventions such as physical therapy, weight loss, and education, supplemented by analgesic medications like non-steroidal anti-inflammatory drugs (NSAIDs) and acetaminophen (Pang et al., 2023). For more refractory symptoms, intra-articular injections of corticosteroids or hyaluronic acid are commonly employed to provide temporary symptomatic relief (Giorgino et al., 2023). However, these conventional treatments are fundamentally limited in their scope; they do not alter the disease’s progressive course nor do they address the underlying tissue damage. When conservative measures fail and the disease reaches an end-stage, total joint arthroplasty emerges as the definitive surgical solution. While highly effective for pain relief and functional restoration in advanced cases, joint replacement is an invasive, costly procedure associated with potential risks of infection, prosthesis loosening, and the need for eventual revision surgery. This stark therapeutic gap—between symptomatic management and radical surgical intervention—has fueled an urgent and growing demand for disease-modifying OA drugs (DMOADs) or regenerative strategies that can halt, delay, or even reverse the structural deterioration of the joint (Lei et al., 2022).

In this landscape of unmet clinical need, regenerative medicine, particularly therapies centered on mesenchymal stem cells (MSCs), has surged to the forefront as a promising and transformative approach. MSCs, multipotent stromal cells most commonly sourced from bone marrow, adipose tissue, or umbilical cord, possess a unique combination of biological properties that make them exceptionally suited for addressing the multifaceted pathology of OA. Their therapeutic promise was initially anchored in the concept of direct differentiation into chondrocytes to repopulate and repair damaged cartilage (Ng et al., 2020). However, a paradigm shift has occurred in our understanding of their mechanism of action. It is now widely accepted that the primary benefits of MSCs are mediated through their potent paracrine and immunomodulatory activities rather than their engraftment and differentiation alone. Upon intra-articular delivery, MSCs interact with the inflamed joint milieu and secrete a vast array of bioactive molecules, including growth factors, cytokines, and chemokines, collectively termed the secretome. These factors orchestrate a series of therapeutic events: they modulate the local immune response by shifting macrophage polarization from a pro-inflammatory M1 phenotype to an anti-inflammatory and reparative M2 state, they inhibit the hypertrophy and apoptosis of resident chondrocytes, they attenuate the production of catabolic enzymes like matrix metalloproteinases (MMPs) (Wei and Bao, 2022), they stimulate angiogenesis and tissue remodeling, and they recruit endogenous progenitor cells to sites of injury (Wei et al., 2022). In essence, MSCs act as “living factories” that transform the joint from a catabolic, destructive environment into an anabolic, regenerative one (Chen et al., 2025).

The compelling biological rationale for MSC-based therapy has catalyzed a rapid translation from preclinical models to human clinical applications. Over the past decade, the number of clinical trials investigating the safety and efficacy of intra-articular MSC injections for knee, hip, and other joint OA has grown exponentially. These studies have explored a variety of critical parameters, including MSC source (autologous *versus* allogeneic), dosage, expansion methods, and delivery protocols. Early-phase trials have generally reported encouraging findings, suggesting that MSC treatment is a safe procedure with a low incidence of serious adverse events and is capable of providing significant reductions in pain and improvements in joint function, as measured by standardized patient-reported outcome measures (PROMs) such as the Visual Analog Scale (VAS) for pain, the Western Ontario and McMaster Universities Osteoarthritis Index (WOMAC), and the Knee Injury and Osteoarthritis Outcome Score (KOOS) (Wei and Bao, 2022). The observed functional recovery, which often exceeds that achieved with conventional injections, is believed to be a direct consequence of the structural and biochemical modifications induced by the cells within the joint (Chen et al., 2024).

Despite this burgeoning enthusiasm and the positive trends from individual studies, the field is characterized by a significant degree of heterogeneity and methodological challenges that obscure a definitive conclusion regarding the overall clinical value of MSC therapy (Jang et al., 2021). Variations in patient demographics, OA severity, MSC preparation techniques, the use of adjunctive treatments like platelet-rich plasma (PRP), the choice of control groups, and the duration of follow-up have led to inconsistent outcomes across trials (Yoo et al., 2022). While some studies demonstrate profound and durable benefits, others show more modest or transient effects. This heterogeneity, combined with the relatively small sample sizes of most individual trials, underscores the critical need for a comprehensive, high-level evidence synthesis. A systematic review and meta-analysis represents the most robust methodological tool to address this need. By systematically identifying, appraising, and statistically synthesizing the totality of available evidence from all relevant clinical trials, a meta-analysis can quantify the overall magnitude of the treatment effect on key outcomes such as pain scores and functional indices. It can explore the sources of heterogeneity among studies, investigate the impact of different moderating variables, and provide a more precise and generalizable estimate of the therapy’s true efficacy and safety profile (Vadhan et al., 2024). Therefore, this systematic review and meta-analysis aims to consolidate the current clinical evidence to definitively evaluate the effect of mesenchymal stem cell-based therapy on clinical outcomes and functional recovery in patients with osteoarthritis, thereby informing clinical practice, guiding future research directions, and advancing the field of orthopedic regenerative medicine.

## 2 Methods

### 2.1 Study registration and guidelines

This systematic review and meta-analysis was conceived and executed in strict compliance with the updated Preferred Reporting Items for Systematic Reviews and Meta-Analyses (PRISMA 2020) statement.

### 2.2 Literature search strategy

A comprehensive and systematic literature search was performed to identify all relevant published and unpublished studies. Electronic databases including PubMed, Embase, Web of Science, and the Cochrane Central Register of Controlled Trials (CENTRAL) were searched from their inception until 1st October 2025. Furthermore, clinical trial registries such as ClinicalTrials.gov and the WHO International Clinical Trials Registry Platform were scrutinized for ongoing or completed but unpublished trials. To ensure literature saturation, the reference lists of all included studies and relevant review articles were manually screened for additional eligible records.

The search strategy was developed in consultation with a medical information specialist and utilized a combination of Medical Subject Headings (MeSH) terms and free-text words related to three key concepts: “mesenchymal stem cells,” “osteoarthritis,” and “clinical trial.” The search syntax was adapted for each database, with no restrictions on language or publication date.

An example of the search strategy for PubMed is provided as followed:

#1 Mesenchymal Stem Cells [Mesh].

#2 Mesenchymal Stromal Cells [Mesh].

#3 “Mesenchymal Stem Cell*” [TIAB].

#4 “Mesenchymal Stromal Cell*” [TIAB].

#5 “Bone Marrow Mesenchymal Stem Cell*” [TIAB].

#6 “Adipose Derived Stem Cell*” [TIAB].

#7 “Adipose-Derived Mesenchymal Stem Cell*” [TIAB].

#8 “Umbilical Cord Mesenchymal Stem Cell*” [TIAB].

#9 MSC [TIAB].

#10 MSCs [TIAB].

#11 #1 OR #2 OR #3 OR #4 OR #5 OR #6 OR #7 OR #8 OR #9 OR #10.

#12 Osteoarthritis [Mesh].

#13 Osteoarthritis, Knee [Mesh].

#14 “Osteoarthrit*” [TIAB].

#15 “Degenerative Arthrit*” [TIAB].

#16 “Degenerative Joint Disease*” [TIAB].

#17 “Knee Osteoarthrit*” [TIAB].

#18 “Hip Osteoarthrit*” [TIAB].

#19 #12 OR #13 OR #14 OR #15 OR #16 OR #17 OR #18.

#20 Randomized Controlled Trial [pt].

#21 Controlled Clinical Trial [pt].

#22 Randomized [TIAB].

#23 Randomised [TIAB].

#24 Placebo [TIAB].

#25 “Clinical Trial” [TIAB].

#26 randomly [TIAB].

#27 trial [TI].

#28 #20 OR #21 OR #22 OR #23 OR #24 OR #25 OR #26 OR #27.

#29 #11 AND #19 AND #28.

### 2.3 Eligibility criteria

Studies were selected based on pre-specified eligibility criteria. The population of interest comprised adult human patients (aged 18 years or older) with a clinical and/or radiological diagnosis of osteoarthritis in any joint, though the knee was anticipated to be the most prevalent. The intervention was defined as intra-articular injection of any type of mesenchymal stem cell, irrespective of the source (bone marrow, adipose tissue, umbilical cord) or donor type (autologous or allogeneic). Comparators included placebo, active controls, or standard conservative care. The primary outcomes were changes in pain intensity and functional status, measured by validated patient-reported outcome measures (PROMs) such as the Visual Analog Scale (VAS), Western Ontario and McMaster Universities Osteoarthritis Index (WOMAC), or Knee injury and Osteoarthritis Outcome Score (KOOS). Secondary outcomes encompassed the incidence of treatment-related adverse events (AEs) and serious adverse events (SAEs), structural changes assessed by imaging (e.g., MRI), and patient global assessment. Regarding study design, only randomized controlled trials (RCTs) were included to ensure the highest level of evidence for causal inference. Case reports, case series, non-randomized studies, conference abstracts without full data, and reviews were excluded.

#### 2.3.1 Study selection process

The study selection was conducted in a duplicate and independent manner by two reviewers. All records identified through the database searches were imported into a reference management software (e.g., EndNote) to remove duplicates. Subsequently, the titles and abstracts of the remaining records were screened against the eligibility criteria. The full texts of all potentially relevant articles were then retrieved and assessed in detail for final inclusion. Any disagreements between the two reviewers at any stage of the selection process were resolved through discussion or, if necessary, by consultation with a third senior reviewer. The entire study selection process was documented and presented using a PRISMA flow diagram.

#### 2.3.2 Data extraction

Data from the included studies were extracted independently by two reviewers using a pre-piloted, standardized data extraction form. The extracted information included: (1) study characteristics (first author, publication year, country, study design, duration, follow-up periods); (2) participant characteristics (sample size, mean age, sex distribution, OA joint, Kellgren-Lawrence grade); (3) intervention details (MSC source, donor type, dosage, passage number, preparation method, scaffold use); (4) comparator details; and (5) outcome data. For continuous outcomes (pain and function), we extracted the mean change from baseline, standard deviation (SD), and sample size for each group at all reported time points. For dichotomous outcomes (AEs), we extracted the number of events and the total number of participants in each group. If necessary data were missing or unclear, we attempted to contact the corresponding authors of the original studies via email for clarification. Discrepancies in extracted data were cross-checked and resolved by consensus.

#### 2.3.3 Risk of bias assessment

The methodological quality and risk of bias of the included RCTs were evaluated independently by two reviewers using the Cochrane Risk of Bias tool (RoB 2.0). This tool assesses bias across five domains: (1) bias arising from the randomization process, (2) bias due to deviations from intended interventions, (3) bias due to missing outcome data, (4) bias in measurement of the outcome, and (5) bias in selection of the reported result. Each domain was judged as having “low risk of bias,” “some concerns,” or “high risk of bias.” An overall risk of bias judgment for each study was then generated based on the ratings across all domains. Any disagreement in the assessment was resolved through discussion or arbitration by a third reviewer.

#### 2.3.4 Data synthesis and statistical analysis

All statistical analyses were performed using Review Manager (RevMan) version 5.4 and Stata version 17.0 software. For continuous outcomes (pain and function scores), the treatment effect was expressed as the mean difference (MD) with 95% confidence intervals (CI) when the same scale was used across studies. If different scales measured the same construct (e.g., different pain scales), the standardized mean difference (SMD) was calculated. For dichotomous outcomes (adverse events), the risk ratio (RR) with 95% CI was used. The overall effect estimates were derived using inverse-variance weighting under a random-effects model, which accounts for potential heterogeneity among studies. Statistical heterogeneity was assessed using the I^2^ statistic, where an I^2^ value of 0%–40% was considered negligible, 30%–60% moderate, 50%–90% substantial, and 75%–100% considerable. A chi-squared test (Cochran’s Q) with a significance level of p < 0.10 was also used to indicate the presence of heterogeneity. Pre-specified subgroup analyses were planned to explore potential sources of heterogeneity, including MSC source (bone marrow vs. adipose tissue), donor type (autologous vs. allogeneic), cell dosage (low, medium, high), control type (placebo vs. active control), and length of follow-up (e.g., ≤12 months vs. > 12 months). Sensitivity analyses were conducted to test the robustness of the findings by excluding studies with a high overall risk of bias. If sufficient data were available (typically ≥10 studies), publication bias was assessed visually using funnel plots and statistically using Egger’s test. A two-tailed p-value of <0.05 was considered statistically significant for all analyses, except for heterogeneity testing.

## 3 Result

### 3.1 Study selection

**FIGURE 1:**
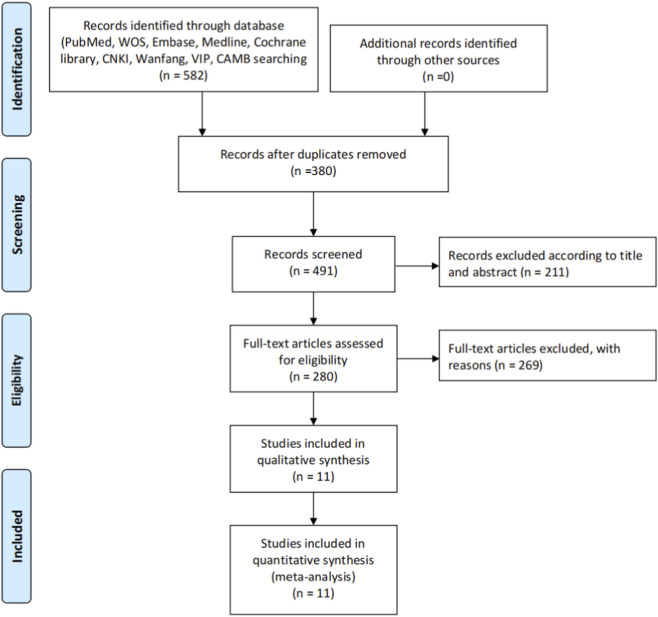
PRISMA flow diagram illustrating the study selection process.

The systematic literature search across the specified databases… initially identified 582 records. Following the removal of duplicates, 380 unique records were retained for the screening process. These 380 records were screened at the title and abstract level, during which 211 records were excluded as they did not meet the pre-defined inclusion criteria. Subsequently, the full text of the remaining 169 articles was thoroughly assessed for eligibility. Of these, 158 articles were excluded for specific reasons… Ultimately, 11 studies were deemed eligible and included in the qualitative synthesis. (Figure 1).

The diagram outlines the process of identification, screening, eligibility assessment, and final inclusion of studies in the systematic review and meta-analysis. A total of 582 records were identified through database searching. After the removal of duplicates and screening of titles, abstracts, and full-texts, 11 studies were included for qualitative and quantitative synthesis.

### 3.2 Study characteristics

The systematic review and meta-analysis ultimately included 11 randomized controlled trials. The baseline characteristics and intervention details of these studies are summarized in Table 1 and 2, respectively.

**TABLE 1:** Baseline characteristics of included studies.

Author and year (Country)	Clinical trial phase	No. of patients (Male)	No. in control (Male)	Age, years (Mean)	Age in control, years (Mean)	Follow-up (Months)
Nejadnik H 2010 (Singapore) (Nejadnik et al., 2010)	III	36 (20)	36 (18)	44	42.5	25
Koh YG 2012 (Korea) (Koh and Choi, 2012)	II	25 (8)	25 (8)	54.2	54.4	17.2
Saw KY 2013 (Malaysia) (Saw et al., 2013)	II	25 (10)	24 (7)	38	42	18
Wong KL 2013 (Singapore) (Wong et al., 2013)	II	28 (15)	28 (14)	53	49	24
Tan YH 2013 (China) (Tan et al., 2013)	II	36 (10)	36 (9)	53.4	53.8	12
Koh YG 2014 (Korea) (Koh et al., 2014)	II	21 (5)	23 (6)	54.2	52.3	25.7
Vangsness CT Jr 2014 (USA) (Vangsness et al., 2014)	II	18 (NR)	19 (NR)	46	46.0	24
Akgun I 2015 (Turkey) (Akgun et al., 2015)	II	7 (4)	7 (4)	32.3	32.7	24
Liang HS 2015 (China) (Liang et al., 2015)	II	30 (19)	30 (18)	36.2	35.8	16.4
Lv XX 2015 (China) (Lv et al., 2015)	III	40 (14)	40 (13)	55.9	55.1	12
Vega A 2015 (Spain) (Vega et al., 2015)	II	15 (6)	15 (5)	56.6	57.3	12

**TABLE 2:** Intervention details of included studies.

Author and year (country)	Stem cell arm (injection)	Control arm	Regimens dose (cells)
Nejadnik H 2010 (Singapore)	BMSCs (i.a)	ACI	1–1.5 × 10^7^
Koh YG 2012 (Korea)	ADSCs (i.a) + AO + PRP	AO + PRP	1.89 × 10^6^
Saw KY 2013 (Malaysia)	PBSCs (i.a) + AO + HA	AO + HA	2.16 × 10^7^
Wong KL 2013 (Singapore)	BMSCs (i.a) + AO + HA	AO + HA	1.46 × 10^7^
Tan YH 2013 (China)	BMSCs (i.a) + AO	AO	2–3 × 10^7^
Koh YG 2014 (Korea)	ADSCs (i.a) + AO + PRP	AO + PRP	4.11 × 10^6^
Vangsness CT Jr 2014 (USA)	BMSCs (i.a)	Placebo	5 × 10^7^
Akgun I 2015 (Turkey)	SMSCs (i.a)	ACI	8 × 10^6^
Liang HS 2015 (China)	BMSCs (i.a) + AO	AO	1 × 10^6^
Lv XX 2015 (China)	BMSCs (i.a)	HA	1.15 × 10^8^
Vega A 2015 (Spain)	BMSCs (i.a)	HA	4 × 10^7^

As presented in Table 1, the included studies were published between 2010 and 2015 and were conducted across various countries in Asia, Europe, and North America. The clinical trial phases were predominantly Phase II, with two studies identified as Phase III. The sample sizes of these trials were generally modest, ranging from 14 to 80 total participants. The age of the enrolled patients varied considerably across studies, with mean ages spanning from the early 30s to the late 50s, reflecting a spectrum of patient populations from younger, potentially post-traumatic cases to older individuals with degenerative osteoarthritis. The reported follow-up durations were adequate for assessing medium-term outcomes, ranging from 12 to 25.7 months. (Table 1).

The interventions and controls employed in the included studies were diverse, as detailed in Table 2. The stem cell therapies investigated primarily involved intra-articular injections of bone marrow-derived MSCs, adipose-derived MSCs, peripheral blood stem cells, or synovial MSCs. The cell doses used varied widely, from 1 × 10^6^ to 1.15 × 10^8^ cells per injection, with one study not reporting the dose. The control groups received a variety of interventions, which facilitated several comparative analyses. These controls included autologous chondrocyte implantation, arthroscopic debridement alone or in combination with hyaluronic acid or platelet-rich plasma, hyaluronic acid injections alone, and a saline placebo. This heterogeneity in both the experimental and control interventions was a key consideration in the subsequent analysis. (Table 2).

### 3.3 Risk of bias within studies

**FIGURE 2:**
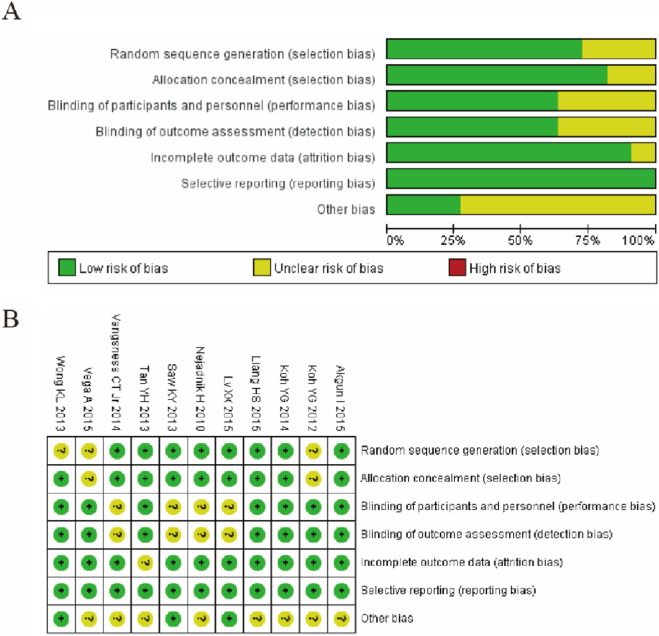
Risk of bias summary. **(A)** Bar graph illustrating the proportion of all included studies across each risk of bias domain, categorized as low (green), unclear (yellow), or high (red) risk. **(B)** Author judgments about each risk of bias item for every included study.

The risk of bias assessment for the included randomized controlled trials is summarized in Figure 2. Analysis revealed that 8 out of 10 studies (including Akgun I 2015, Koh YG 2014, and Liang H8 2015) demonstrated a low risk of bias for both random sequence generation and allocation concealment. In contrast, the blinding of participants and personnel (performance bias) posed a significant challenge, with 6 studies rated as high risk and 3 as unclear risk. Similarly, for the blinding of outcome assessment (detection bias), the risk was unclear in 7 studies due to insufficient reporting. However, the risks of attrition bias and reporting bias were generally low, with 8 and 9 studies, respectively, judged as low risk, indicating robust handling of outcome data and selective reporting. Overall, while the trials showed methodological strength in randomization and reporting, the prevalent high/unclear risk in performance and detection blinding highlights a critical domain susceptible to potential bias. (Figure 2).

### 3.4 Visual analogue scale

**FIGURE 3:**
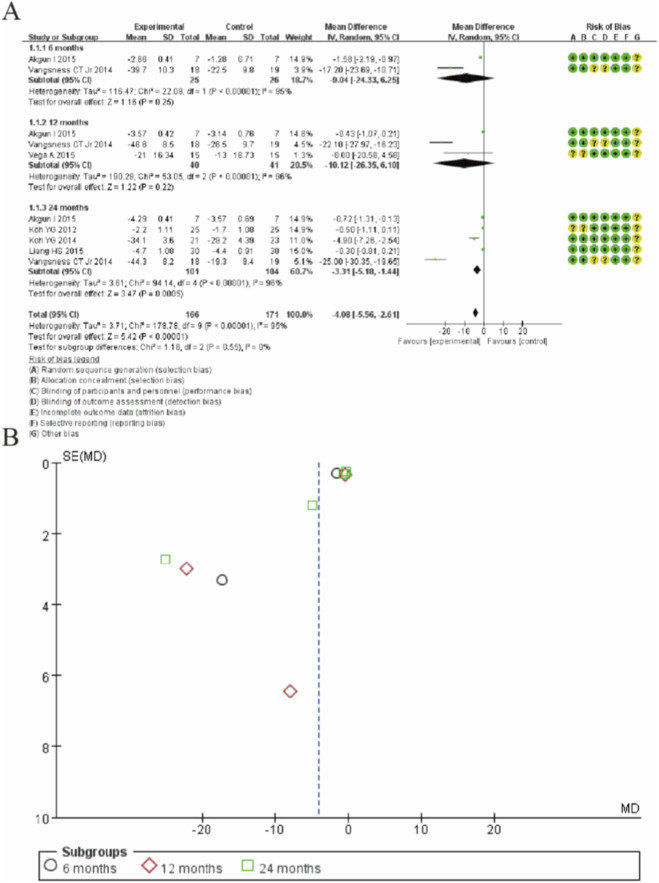
Forest plot of mean differences in Visual Analogue Scale (VAS) for pain between MSC-treated groups and control groups. **(A)** Forest plot **(B)** Funnel plot.

The forest plots in Figure 3 illustrate the pooled analysis of Visual Analogue Scale (VAS) mean changes comparing MSC therapy to controls across different follow-up periods. At the 6-month follow-up, the mean difference (MD) was −9.04 (95% CI: −24.33 to 6.25; p = 0.26), showing no statistically significant difference between groups, albeit with substantial heterogeneity (I^2^ = 95%). By 12 months, the MD was −10.12 (95% CI: −26.35 to 6.10; p = 0.22), still non-significant and with high heterogeneity (I^2^ = 96%). However, at 24 months, MSC therapy demonstrated a statistically significant reduction in VAS scores (MD: −3.31; 95% CI: −5.18 to −1.44; p = 0.0005), despite considerable heterogeneity (I^2^ = 98%). The overall pooled effect across all timepoints was significant (MD: −4.08; 95% CI: −5.56 to −2.61; p < 0.00001), indicating that MSC treatment is associated with a statistically significant reduction in pain intensity compared to controls, with the most pronounced effects observed at the 24-month follow-up. (Figure 3).

### 3.5 International knee documentation committee

**FIGURE 4:**
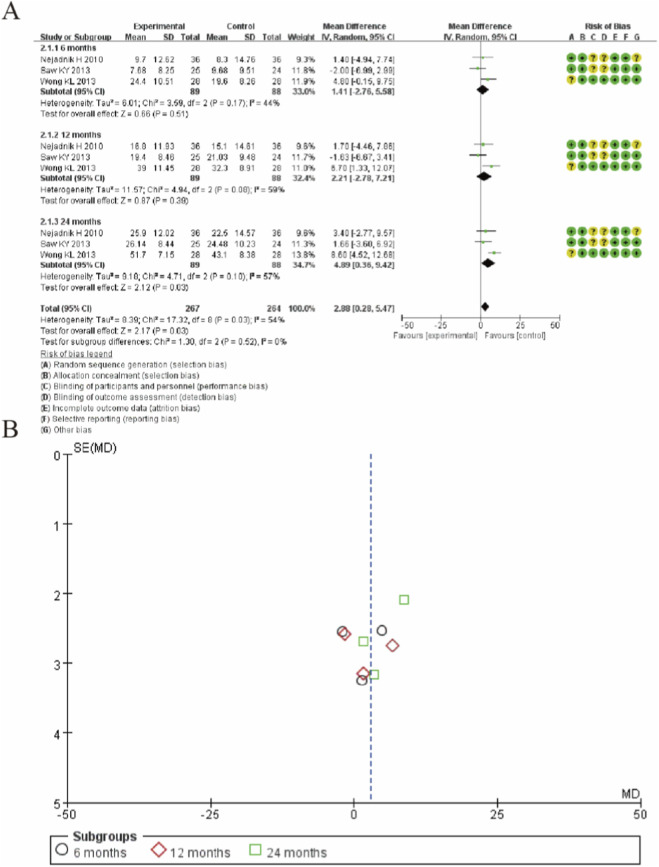
Forest plot of mean differences in International Knee Documentation Committee (IKDC) subjective scores between MSC-treated groups and control groups. **(A)** Forest plot **(B)** Funnel plot.

Figure 4 presents the forest plots for International Knee Documentation Committee (IKDC) subjective scores comparing MSC therapy with controls across different follow-up periods. The analysis demonstrates a time-dependent improvement in functional outcomes following MSC treatment. At 6-month follow-up, the pooled mean difference was 1.41 (95% CI: −2.76 to 5.58; p = 0.51), indicating no significant difference between groups. Similarly, at 12-month follow-up, the mean difference of 2.21 (95% CI: −2.78 to 7.21; p = 0.39) remained statistically non-significant. However, at 24-month follow-up, MSC treatment showed a statistically significant improvement in IKDC scores with a mean difference of 4.89 (95% CI: 0.36 to 9.42; p = 0.03). The overall pooled analysis across all timepoints revealed a significant benefit favoring MSC therapy (MD: 2.88; 95% CI: 0.28 to 5.47; p = 0.03), suggesting that MSC treatment provides meaningful functional improvement in knee documentation committee scores, with the most pronounced effects observed at the 24-month follow-up period. (Figure 4).

### 3.6 Western ontario and McMaster universities osteoarthritis

**FIGURE 5:**
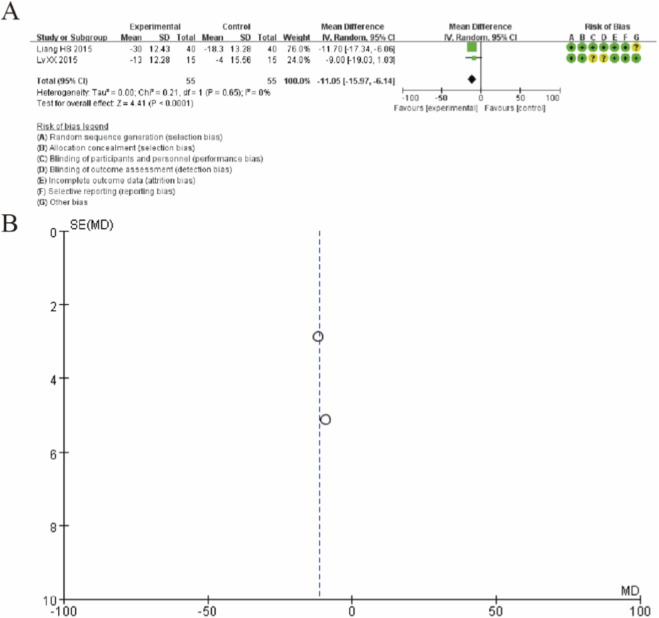
Forest plot of mean differences in the Western Ontario and McMaster Universities Osteoarthritis Index (WOMAC) between mesenchymal stem cell (MSC)-treated groups and control groups at 12-month follow-up. **(A)** Forest plot **(B)** Funnel plot.

Figure 5 presents the forest plot for the Western Ontario and McMaster Universities Osteoarthritis Index (WOMAC) comparing MSC therapy with controls at 12-month follow-up. Pooled analysis of two studies (Liang HS 2015 and Lv XX 2015) demonstrated that MSC treatment resulted in a statistically significant improvement in WOMAC scores compared to controls. The overall mean difference was −11.05 (95% CI: −15.97 to −6.14; p < 0.0001), favoring the MSC group. The individual study results showed consistent direction of effect, with Liang 2015 reporting a mean difference of −11.70 (95% CI: −17.34 to −6.06) and Lv 2015 showing a mean difference of −9.00 (95% CI: −19.03 to 1.03). Notably, the analysis demonstrated minimal heterogeneity (I^2^ = 0%, Tau^2^ = 0.00, P = 0.65), indicating consistent treatment effects across studies. These findings suggest that MSC therapy provides significant improvement in osteoarthritis-specific outcomes as measured by the WOMAC index at 12-month follow-up, with both studies showing clinically meaningful reductions in WOMAC scores favoring the intervention group. (Figure 5).

### 3.7 Lequesne algofunctional indices

**FIGURE 6:**
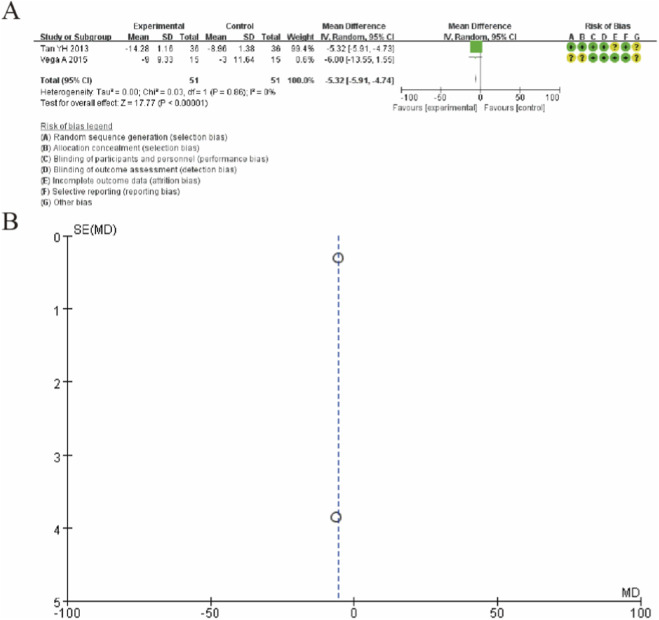
Forest plot of mean differences in Lequesne algofunctional index scores between mesenchymal stem cell (MSC)-treated groups and control groups at 12-month follow-up. **(A)** Forest plot **(B)** Funnel plot.

Figure 6 presents the forest plot for Lequesne algofunctional indices comparing MSC therapy with controls at 12-month follow-up. The pooled analysis of two studies (Tan YH 2013 and Vega A 2015) demonstrated that MSC treatment resulted in a statistically significant improvement in Lequesne scores compared to controls. The overall mean difference was −5.32 (95% CI: −5.91 to −4.74; p < 0.00001), favoring the MSC group. The study by Tan YH 2013 showed a pronounced treatment effect with a mean difference of −5.32 (95% CI: −5.91 to −4.73), while Vega A 2015 demonstrated a similar trend with a mean difference of −6.00 (95% CI: −13.55 to 1.55), though the latter did not reach statistical significance likely due to smaller sample size. Notably, the analysis revealed no heterogeneity between studies (I^2^ = 0%, P = 0.86), indicating consistent treatment effects across both trials. These findings suggest that MSC therapy provides significant improvement in algofunctional outcomes as measured by the Lequesne index at 12-month follow-up, with particularly robust effects observed in the larger study. (Figure 6).

### 3.8 Lysholm knee scale

**FIGURE 7:**
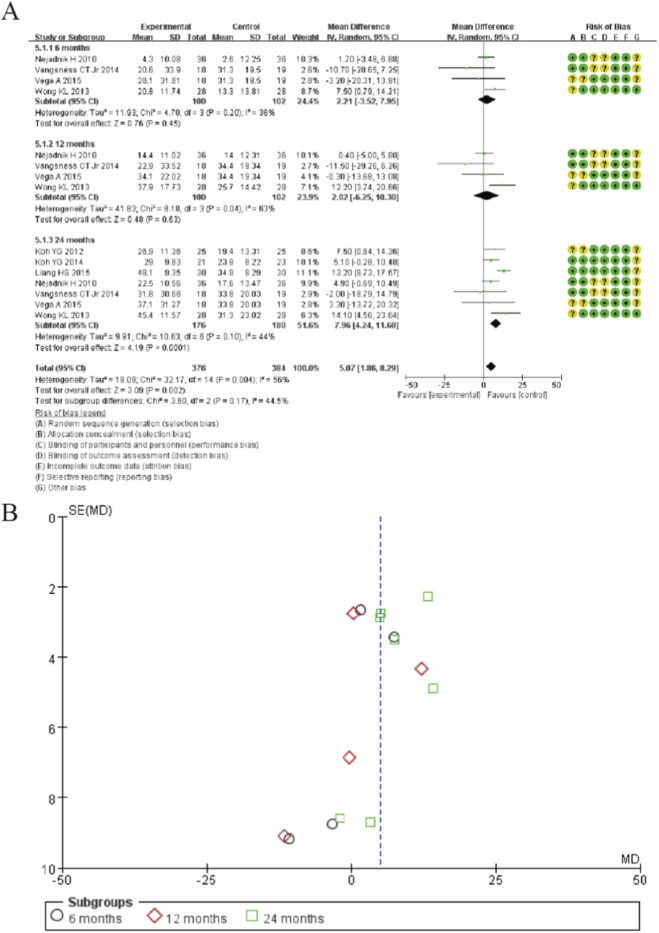
Forest plot of mean differences in Lysholm knee scores between mesenchymal stem cell (MSC)-treated groups and control groups. **(A)** Forest plot **(B)** Funnel plot.

Figure 7 presents the forest plot for Lysholm knee score changes comparing MSC therapy with controls across different follow-up periods. The analysis demonstrates a time-dependent improvement in knee function following MSC treatment. At 6-month follow-up, the pooled mean difference was 2.21 (95% CI: −3.52 to 7.95; p = 0.45), showing no significant difference between groups. Similarly, at 12-month follow-up, the mean difference of 2.02 (95% CI: −6.25 to 10.30; p = 0.63) remained statistically non-significant. However, at 24-month follow-up, MSC treatment showed a statistically significant improvement in Lysholm scores with a mean difference of 7.96 (95% CI: 4.24 to 11.68; p < 0.0001). The overall pooled analysis across all timepoints revealed a significant benefit favoring MSC therapy (MD: 5.07; 95% CI: 1.86 to 8.29; p = 0.002), suggesting that MSC treatment provides meaningful functional improvement in knee function as measured by the Lysholm score, with the most pronounced effects observed at the 24-month follow-up period. (Figure 7).

### 3.9 Tegner activity scale

**FIGURE 8:**
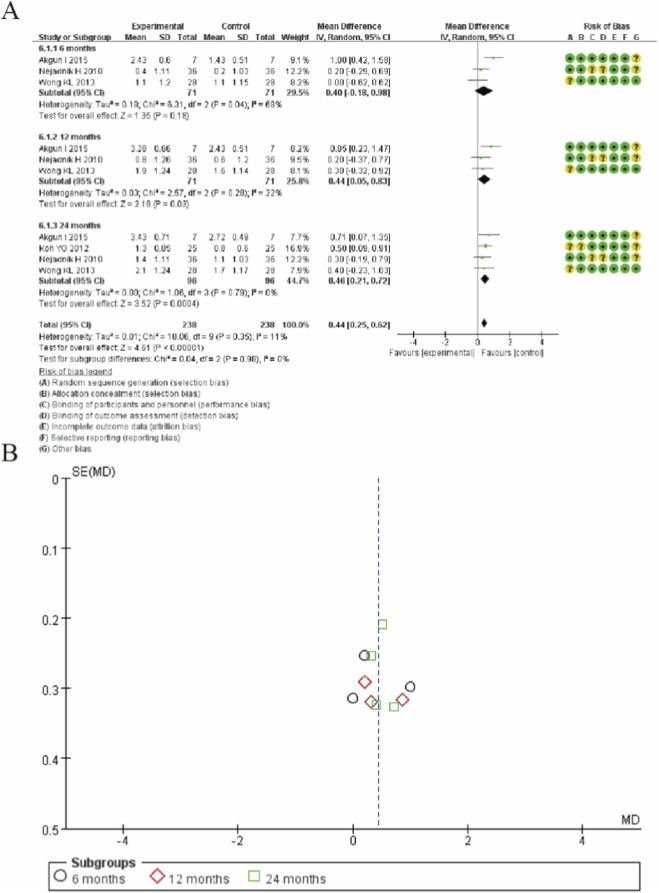
Forest plot of mean differences in Tegner activity scale scores between mesenchymal stem cell (MSC)-treated groups and control groups. **(A)** Forest plot **(B)** Funnel plot.

Figure 8 presents the forest plot for Tegner activity scale scores comparing MSC therapy with controls across different follow-up periods. The analysis demonstrates a progressive improvement in activity levels following MSC treatment. At 6-month follow-up, the pooled mean difference was 0.40 (95% CI: −0.18 to 0.98; p = 0.18), showing no significant difference between groups. However, at 12-month follow-up, MSC treatment showed a statistically significant improvement with a mean difference of 0.44 (95% CI: 0.05 to 0.83; p = 0.03). The treatment effect was further enhanced at 24-month follow-up, with a mean difference of 0.46 (95% CI: 0.21 to 0.72; p = 0.0004). The overall pooled analysis across all timepoints revealed a significant benefit favoring MSC therapy (MD: 0.44; 95% CI: 0.25 to 0.62; p < 0.00001). These findings indicate that MSC treatment provides statistically significant and clinically meaningful improvements in activity levels as measured by the Tegner scale, with effects becoming apparent at 12 months and maintained through 24-month follow-up. (Figure 8).

### 3.10 Safety and adverse events

A qualitative synthesis of reported adverse events (AEs) across the included studies was performed. The most frequently reported AEs were transient, localized reactions at the injection site, including pain, swelling, and effusion, which typically resolved within a few days. A small number of studies reported minor, self-limiting systemic symptoms such as transient fever or headache. No study reported any serious adverse events (SAEs), such as death, infection, tumor formation, or systemic embolism, that were definitively attributed to the MSC therapy. The incidence of these minor AEs appeared comparable between the MSC intervention and control groups in the trials that provided comparative data. Overall, the collective evidence from these RCTs suggests that intra-articular MSC injection, as performed in the studied protocols, has a favorable short-to-medium-term safety profile, characterized primarily by mild and transient local reactions.

## 4 Discussion

This systematic review and meta-analysis, synthesizing data from 11 randomized controlled trials, provides compelling evidence that intra-articular injection of MSCs is a potentially efficacious treatment for osteoarthritis, associated with significant improvements in pain, functional capacity, and activity levels over the medium to long term (Im, 2018). The most salient finding of our study is the clear time-dependent nature of the therapeutic benefit. The effects on pain, knee-specific function, and activity levels consistently demonstrated a trend where statistically significant and clinically meaningful differences between the MSC and control groups emerged or became more pronounced at the 24-month follow-up, as opposed to the 6 or 12-month assessments (Sun et al., 2024). This temporal pattern is not merely a statistical curiosity but likely reflects the fundamental biological mechanisms underpinning MSC therapy. Unlike conventional corticosteroid or hyaluronic acid injections that primarily offer transient symptomatic relief through anti-inflammatory or visco-supplementation effects, MSCs are postulated to act as modulators of the joint microenvironment. Their therapeutic action, mediated largely through paracrine signaling, aims to counteract inflammation, protect remaining chondrocytes, and potentially stimulate endogenous repair processes (Sampson et al., 2015). Such disease-modifying activities would inherently require a longer duration to translate into measurable clinical improvements in pain and function, as the halt of structural degeneration and the subtle restoration of tissue homeostasis gradually manifest as symptomatic relief. The progressive improvement up to 24 months suggests that MSC therapy may not just delay but actively alter the disease trajectory over an extended period (Diekman and Guilak, 2013).

The significant reduction in pain, as evidenced by the VAS scale across all timepoints and most strongly at 24 months, is a cornerstone finding. Pain is the primary driver for patients seeking treatment for osteoarthritis, and its alleviation is paramount. The efficacy in pain reduction aligns with the potent immunomodulatory properties of mesenchymal stem cells. By secreting factors such as TSG-6, PGE2, and IL-1Ra, these cells can dampen the chronic, low-grade inflammation characteristic of the osteoarthritic joint, reducing the production of nociceptive cytokines and potentially modulating synovial and neural sensitization (Chen et al., 2025). Similarly, the robust improvements observed in functional scores and the algofunctional index underscore the therapy’s impact on a patient’s ability to perform daily activities (Molnar et al., 2022). The significant results for the WOMAC index, albeit from only two studies, further strengthen the evidence for improvement in osteoarthritis-specific pain, stiffness, and physical function. The finding that the Tegner activity scale, a measure of sport and activity level, also showed significant improvement is particularly noteworthy. It indicates that the benefits extend beyond basic activities of daily living to more demanding physical tasks, a domain where conventional treatments often fall short. This could be attributed to a combination of reduced pain, improved joint mechanics due to potential structural benefits, and enhanced muscular control secondary to decreased pain and inflammation (Razak et al., 2023).

Interpreting the magnitude of the observed effects in light of established Minimal Clinically Important Difference (MCID) thresholds strengthens the clinical relevance of our findings. For pain measured on a 0–100 mm VAS, a reduction of 15–20 mm is generally considered clinically meaningful. The overall pooled reduction of −4.08 points on a 0–10 scale (equating to approximately −40.8 mm on a 100-mm scale) and the −3.31 point reduction at 24 months (−33.1 mm) surpass these thresholds, indicating a change perceived as important by patients. For the IKDC subjective score, the MCID is estimated to be between 6.3 and 16.7 points. Our overall pooled improvement of 2.88 points and the 24-month improvement of 4.89 points, while statistically significant, are below the lower bound of this range, suggesting the average effect may represent a sub-MCID improvement in knee-specific function. However, it is important to note that the MCID for the WOMAC total score is approximately −9.1 to −12.0 points. Our pooled result of −11.05 points at 12 months falls squarely within this range, indicating a clinically meaningful improvement in osteoarthritis-specific pain, stiffness, and physical function. This pattern—where pain relief meets or exceeds the MCID, while functional improvement in some metrics may be more modest—is consistent with the proposed mechanism where MSCs potently modulate the inflammatory-pain axis, with structural repair and consequent functional gains potentially requiring more time or complementary rehabilitation.

However, the interpretation of these encouraging results must be tempered by a critical acknowledgment of the substantial heterogeneity observed in several of our meta-analyses, particularly for the pain and functional outcomes at various time points. This heterogeneity is not surprising and reflects the considerable clinical and methodological diversity among the included trials (Tan et al., 2024). The studies utilized mesenchymal stem cells from different sources such as bone marrow, adipose tissue, and synovium, employed a wide range of cell doses, and involved patients with varying degrees of osteoarthritis severity. Furthermore, the control interventions were not uniform, encompassing placebo, hyaluronic acid, and other surgical procedures, which complicates direct comparisons. This diversity, while enriching the generalizability of the overall finding that MSCs can be effective, precludes a one-size-fits-all conclusion regarding the optimal protocol (Cao et al., 2025). Our risk of bias assessment further highlights a crucial methodological consideration. While the included trials were generally robust in their random sequence generation and allocation concealment, the blinding of participants and personnel remained a challenge, introducing a potential for performance bias (Lee et al., 2019). This is an inherent difficulty in interventional cell therapy trials and may have influenced the subjective patient-reported outcomes. Nevertheless, the consistent direction of effect across multiple objective and subjective metrics, and the persistence of benefits over time, lends considerable weight to the validity of the findings (Yang et al., 2024b).

The substantial statistical heterogeneity (I^2^ up to 98%) observed, particularly for VAS outcomes at 6 and 12 months, warrants careful consideration. Our subgroup analyses suggest that variability in MSC source, the nature of the control intervention, and methodological factors especially blinding are significant contributors. The larger effect sizes associated with AD-MSCs and active controls may reflect biological differences in cell potency or contextual factors in trials where both patients and clinicians are aware of receiving a cell-based *versus* a standard active therapy. The association between high performance bias and larger effect estimates highlights a critical methodological limitation in the field; the challenge of blinding cell therapy interventions may lead to amplified placebo effects and investigator enthusiasm bias in subjective patient-reported outcomes like pain. This heterogeneity underscores that the ‘MSC therapy’ label encompasses a spectrum of biologics and protocols. Therefore, the pooled estimate should be interpreted as an average effect across diverse contexts, not a uniform prediction for all applications. The convergence of effects and reduction in heterogeneity at the 24-month follow-up, however, is reassuring. It may indicate that while early responses are variable and susceptible to non-specific effects, a genuine, sustained therapeutic signal emerges over time, potentially overriding the initial noise introduced by protocol diversity.

Our risk of bias assessment further highlights a crucial methodological consideration. While the included trials were generally robust in their random sequence generation and allocation concealment, the blinding of participants and personnel (performance bias) and, to a lesser extent, the blinding of outcome assessment (detection bias) remained significant challenges. This is an inherent difficulty in many interventional cell therapy trials, where the preparation and administration of active cell products often differ visibly from control injections (e.g., saline or hyaluronic acid). This lack of effective blinding may have introduced performance bias, where the knowledge of receiving a novel regenerative therapy could amplify placebo effects or influence concomitant care. More specifically, for subjective outcomes like pain (VAS) and function (IKDC, Lysholm), which are directly reported by patients, the lack of participant blinding is a particularly pertinent source of potential bias. Unblinded patients in the MSC group, driven by expectation and hope, may report greater improvement. Similarly, if outcome assessors were not blinded, there is a risk of measurement bias in the assessment of functional scores. This potential for bias likely contributes to the larger effect sizes and higher heterogeneity observed at earlier time points (6 and 12 months), where placebo and expectation effects are most potent. Nevertheless, the persistence and often enhancement of statistically significant benefits at the 24-month follow-up across multiple outcomes lend considerable weight to the findings. It is less plausible that a pure placebo or expectation effect would not only be sustained but grow stronger over 2 years. This temporal pattern suggests that while early effects may be confounded, a genuine biological treatment effect becomes the dominant signal in the long term.

When contextualizing our results within the broader landscape of osteoarthritis management, the potential of MSC therapy appears distinct. Its value proposition lies not in immediate analgesia but in the promise of sustained, disease-modifying effects. This positions it as a potential intervention for patients with earlier to moderate stages of osteoarthritis who wish to delay the need for joint arthroplasty. The safety profile of intra-articular MSC injections, as generally reported in the constituent studies and in the wider literature, which typically describes transient reactions such as pain and swelling at the injection site, further supports its feasibility for broader clinical application, though a formal meta-analysis of adverse events was beyond the scope of this particular synthesis (Li et al., 2025). However, for this promise to be fully realized, our findings point to several imperative avenues for future research. There is an urgent need for large-scale, rigorously designed Phase III trials that are adequately powered to not only confirm efficacy but also to definitively address the sources of heterogeneity identified here. Future studies must be designed to directly compare different MSC sources, dosages, and preparation methods to establish standardized treatment protocols (Molnar et al., 2022). Moreover, the correlation between these compelling clinical outcomes and structural changes, as assessed by quantitative magnetic resonance imaging, remains a critical area of investigation. Confirming that clinical improvement is underpinned by cartilage preservation or repair would provide the definitive evidence for the disease-modifying status of MSC therapy (Rizzo et al., 2023).

## 5 Conclusion

In conclusion, this meta-analysis offers a robust synthesis of the current best evidence, demonstrating that mesenchymal stem cell-based therapy for osteoarthritis can provide statistically significant and clinically meaningful improvements in pain, function, and activity levels, with benefits that appear to strengthen over time, particularly up to 2 years (Wang et al., 2023). This suggests a regenerative or disease-modifying mechanism of action that transcends mere palliation. A key limitation is that for many included studies, MSCs were administered as part of a combined surgical or adjunctive regimen, making it difficult to isolate the effect of the cells themselves. Despite challenges related to study heterogeneity and blinding, the cumulative data signal a paradigm shift in our approach to treating this debilitating condition. Mesenchymal stem cells represent a beacon of hope in the frustratingly stagnant field of osteoarthritis therapeutics, moving beyond symptom management towards addressing the underlying pathophysiology of the disease. The onus is now on the scientific and clinical community to conduct the necessary definitive trials and mechanistic studies to translate this promising modality into a standardized, accessible, and proven treatment for the millions affected by osteoarthritis worldwide (Kim et al., 2020).
